# Dataset of 69 German COVID-19 Snapshot Monitoring (COSMO) surveys on pandemic attitudes, feelings and behaviours

**DOI:** 10.1038/s41597-025-05275-3

**Published:** 2025-06-23

**Authors:** Cornelia Betsch, Sarah Eitze, Lars Korn, Lisa Felgendreff, Anne-Sophie Tänzer, Philipp Sprengholz

**Affiliations:** 1 Institute for Planetary Health Behaviour, University of Erfurt, 99089 Erfurt, Germany; 2 Bernhard Nocht Institute for Tropical Medicine, 20359 Hamburg, Germany; 3 University of Bamberg, 96047 Bamberg, Germany

**Keywords:** Human behaviour, Communication, Risk factors, Decision making

## Abstract

During a pandemic, knowledge, risk perceptions, trust in institutions and attitudes towards public health measures influence protective behaviours and mental health. The COVID-19 Snapshot Monitoring (COSMO) project collected psychosocial data on pandemic-related attitudes, feelings and behaviours from representative samples in Germany. In sixty-nine cross-sectional online surveys conducted between 03.03.2020 and 29.11.2022, *N* = 69,013 individuals were assessed. Our 332 variables show how COVID-19 was perceived (e.g., symptoms, risk perceptions), which behaviours were exhibited (e.g., mask wearing, keeping distance, being vaccinated, meeting other people), what attitudes and beliefs people held (e.g., towards vaccination, conspiracy beliefs, pandemic fatigue), which information sources they used and trusted, how their mental health was affected (e.g., worries, wellbeing, resilience) and what consequences the pandemic had for people (e.g., financial losses, alcohol consumption). Variables are available for at least five waves (i.e., roughly 5,000 participants), some variables are available for all waves. The data allow tracing population-level changes in pandemic perceptions and actions, assess the relationships between determinants and behaviours, and help prepare for future crises.

## Background & Summary

The COVID19-Snapshot Monitoring (COSMO) is a research project that aimed to gain insights into how the German population perceived the COVID-19 pandemic, the political measures taken to contain the pandemic and its psychosocial impact on everyday life in Germany between March 2020 and November 2022. The collected data were intended to provide political stakeholders, the media and the population with helpful knowledge about the psychological aspects of the pandemic and to prevent misinformation (e.g., regarding the acceptance of political measures) and knee-jerk reactions based on unfounded assumptions about how members of the public feel or behave^1,2^. In addition, the pandemic yielded a unique opportunity for social and behavioural scientists to study human behaviour in a crisis. The evidence gathered can support future pandemic preparedness^3^. In addition, the presented data can help evaluate some of the predictions made by behavioural science and thus also add to the discussion of behavioural science’s usefulness in guiding crisis responses^4^. For example, in the COSMO study, efforts were made to contextualise behaviours and public health measures that were heavily debated in the media, such as mask wearing^5^, rapid testing^6^ and nationwide lockdowns^7^. Future users can make use of the data by assessing perceptions in relations to such policy changes (e.g. as collected by the Oxford Covid-19 Government Response Tracker (OxCGRT^8^)).

## Methods

A methodological preprint published at the survey’s outset^9,10^ was utilised to establish a standard protocol, which was recommended by WHO Europe^11^ for use as a WHO-tool^12^ and adopted by about 40 countries (e.g., Denmark^13^, Spain^14^, Finland^15^, Iran^16^).

### Ethical review

Ethical clearance was obtained from the University of Erfurt’s institutional review board (#20200302/2020831/20200501). It was approved that the study follows the ethical guidelines of the University of Erfurt, that the safety and well-being of the subjects was ensured at any time, that there were no ethical concerns for the recruitment of the participants, the implementation of the study design or the subsequent data analysis and processing, and that the undertaken measures safeguard the ethical standards as proposed by the DGPs (German Psychological Society). Participants gave informed consent to take part in the study voluntarily, with the understanding that their data would be used solely for scientific purposes and for supporting public health communication efforts. They were informed that the anonymized data would be stored permanently and may be shared with other researchers for future scientific use. Informed consent was obtained through an online consent form, presented prior to the start of the actual study: Participants had to actively agree to the terms and confirm their understanding before proceeding to the questionnaire. To ensure privacy and data protection, all data was collected in an anonymized way. No personally identifying information (such as names or contact details) was collected or stored.

### Sampling method

The project comprised 69 cross-sectional surveys conducted in Germany between March 2020 and November 2022. While the surveys were performed weekly to biweekly in the first year of the project, the frequency of survey administration decreased later. Each survey took between 20 and 30 minutes and was completed by approximately 1,000 individuals (*N* = 69,013). The data provider Bilendi GmbH recruited and compensated the respondents. Data collection typically took place between Tuesday afternoon and Wednesday at midnight during the data collection weeks. Such a short data collection period was chosen as at some timepoints during the pandemic COVID-19 cases, decisions and media debates about political measures were advancing quickly. As the survey aimed to provide a rather exact snapshot, the data were collected within roughly 36 hours. The sampling was quota-representative in terms of age (18–74 years of age) and gender (crossed), as well as the German federal states, according to census data^17^. Because the quota sample was based on the German census data, which did not include more than two genders at the time, only men and women were sampled by quota. For a better impression of the sample, Table 1 provides the quotas in the sample in comparison to the available census data. As can be seen, the sample was higher educated than the German population, but please note that this was not a stratification criterion. When quotas were completed, participants were screened out after providing their quota-relevant demographics.

**Table 1:** Representativeness of the sample as compared to 2011 census data.

				Census	Full sample*
Federal state	Baden-Wuerttemberg			13%	12.1% (n = 8373)
	Bavaria			15%	15.3% (n = 10542)
	Berlin			4%	4.5% (n = 3113)
	Brandenburg			3%	3.1% (n = 2168)
	Bremen			1%	0.8% (n = 567)
	Hamburg			2%	2.3% (n = 1612)
	Hesse			7%	7.5% (n = 5161)
	Mecklenburg-Western Pomerania			2%	2.1% (n = 1439)
	Lower Saxony			10%	9.5% (n = 6546)
	North Rhine-Westphalia			22%	21.9% (n = 15145)
	Rhineland-Palatinate			5%	4.9% (n = 3396)
	Saarland			1%	1.3% (n = 878)
	Saxony			5%	5.3% (n = 3630)
	Saxony-Anhalt			3%	2.9% (n = 1992)
	Schleswig-Holstein			3%	3.6% (n = 2491)
	Thuringia			3%	2.8% (n = 1960)
Education**	High			30%	56% (n = 38651)
	Medium			28%	33.3% (n = 23012)
	Low			37%	10.7% (n = 7350)
		**Female gender**		**Male gender**	
		**Census**	Full sample*	**Census**	Full sample*
Age	18 to 29	18.4%	19.3% (n = 6737)	19.8%	18.6% (n = 6336)
	30 to 49	37.9%	37.8% (n = 13212)	38.5%	37.9% (n = 12901)
	50 to 64	27.6%	27.7% (n = 9697)	27.5%	28.1% (n = 9570)
	65 to 74	16.1%	15.3% (n = 5351)	14.3%	15.3% (n = 5194)

*Note*: N = 69,013. *Percentages may vary within the single waves. **The sampling was based to reach representativeness for age x gender, federal state; education was not a stratification criterion but is reported here to point to the bias towards higher education.

### Measurements

Table 2 provides an overview of constructs available in the dataset. Note that for most constructs there is a set of items or a validated scale available. The questionnaire covered socio-demographic information; the monitoring of risk perceptions^18^; emotional responses to the pandemic^19–21^; self-efficacy^22,23^, trust in institutions^24^; adherence to protective measures; a range of concerns; general burden and wellbeing; vaccination-related attitudes, intentions and behaviours; attitudes towards upcoming and implemented policies and conspiratorial tendencies^25,26^. All original questionnaires are available online (https://osf.io/w38cn/). Note that the single questionnaires may contain variables not included in the dataset, because there were not assessed at least five times, and this was the criterion for including the variables in this data set.

**Table 2:** Summarised overview of constructs available in the dataset.

Type of variables	Variables
Socio-demographic variables	Age, gender, education, federal state, being chronically ill, having children, household size, marital status, community size, employment, having a system-relevant job, belonging to a COVID-19 risk group, political preferences, belonging to a minority, migration background, income, opportunity for home office.
Attitudes, knowledge and beliefs	Self-efficacy, vaccination readiness* (self and children), conspiracy beliefs*, vaccine preferences, trust in several institutions (e.g., national health authorities, local and national government, science, doctors, and the media), knowledge (e.g., about the ways of infection, the availability of treatment and prevention measures, and measures that are required by law), the perception of the virus and the disease (risk perceptions, symptoms experienced when infected, knowing someone who had died from COVID-19, acceptance of measures (general and specific measures, e.g., wearing masks in schools and the restriction of civil liberties), the perceived effectiveness of measures (e.g., mask wearing and lockdowns), reactance due to pandemic measures, pandemic fatigue*.
Self-reported behaviours	Vaccination (self and children, including boosters), recommended protective behaviours (e.g., mask wearing, airing the room, keeping distance, avoiding close contacts, using a tracing app, disinfecting one’s hands, using rapid tests and self-quarantining), risk behaviour (e.g., meeting people), prepping behaviour (e.g., storing medicine and food).
Mental health and mental resources	Worries, resilience*, wellbeing*, having been diagnosed with a mental illness, religiosity, satisfaction with life*, feeling burdened.
Health information	Frequency of using certain information sources, trust in information sources, feeling informed, frequency of searching for information.
Consequences of the pandemic	Financial losses, families spending more time with one another, drug consumption.

*Note*: For all variables and the exact wording of all variables, see data legend (10.23668/psycharchives.15213). Constructs with asterisk (*) were assessed with validated scales.

Due to extreme time pressure, high workload and limited resource there was no formalized advisory process regarding which variables to assess or to drop from the study; topics were gathered from press briefings and the news, interactions with study partners, meetings with government and administration representatives and discussions with other scientists (see acknowledgements). Thus, the process can be described as rather bottom up, problem-oriented and use-inspired, partially due to the speed of changes happening during the pandemic as well as the short intervals between the data collections. Most variables and items were therefore newly constructed as they responded to events or behaviours that became important over the course of the pandemic. Yet, there are several validated scales that were used repeatedly, namely the 5 C vaccination readiness^27^, conspiracy beliefs^25,26^, pandemic fatigue^28^, wellbeing (WHO5^20,21^), resilience^29^ and satisfaction with life^30^. Detailed information about the items included in the data set, their measurement and English translations are presented in the codebook, which is also available in the repository^31^.

## Data Records

The dataset is available at PsychArchives^31^ and provides as a csv and Excel file. For use in R we suggest using the csv file. All survey measures that were assessed in at least five surveys were considered for publication. This selection was made to create larger samples per potential question and identify themes and variables that were relevant for a substantial period of time. Some variables were assessed only once or belonged to single experiments and are not presented here. The data set contains a column for each measure and a row for each participant. Two columns identify the time point of the sampling in the form of the chronological number of the survey (TIME, numerical order from 1 to 70; the survey TIME = 36 was used for a related longitudinal follow-up survey, and is not included in the cross-sectional dataset described here), as well as the specific survey date (TIME_NOMINAL, ranging from 03.03.20 to 29.11.22; indicating the day on which the 36-hour data collection period began). Note that there is an additional time-point indicated by TIME = 71 from September 2023 that only includes socio-demographics, vaccination readiness and intention to get vaccinated. We do not consider this a full wave but report this follow up for completeness.

## Technical Validation

The data provider guaranteed the data quality. The company regularly checked the quality of participants’ answers in its own surveys and removes bots and unreliable participants. We further ensured the cross-sectional nature of the data collection by not inviting participants who had participated in one of the previous 20 weeks to reduce conditioning effects. An additional check was also run in R, which identified and removed duplicates within a wave and in relation to previous waves for which a participant would still be blocked. Other than that, no validation or clean-up processes were implemented. Subsequent users of the dataset can further clean the data (e.g., by removing participants with fast or slow survey durations or whose answer patterns indicate inattentive responding).

## Usage Notes

The presented dataset is unique, as it covers behavioural data from the pandemic from its beginning across almost three important and eventful years. The data collections were highly adaptive, which led to the rapid generation of items. While this confers the advantage that the themes and topics that were brought up by the media, society or politics could be integrated swiftly, creating a thorough theoretical foundation for each item or research question was not always possible. In addition, the large quantity of data allows us to explore a nearly endless number of relationships. While we believe that exploring the data may be valuable, confirmative research will require preregistration. We therefore suggest preregistering the research question and hypotheses at hand.

The data is made available for public use (Sharing level 0), allowing open and immediate access (https://psycharchives.org/en/about#sharing_levels).

### COVID-19 Behavioural data dashboard

**Fig. 1:**
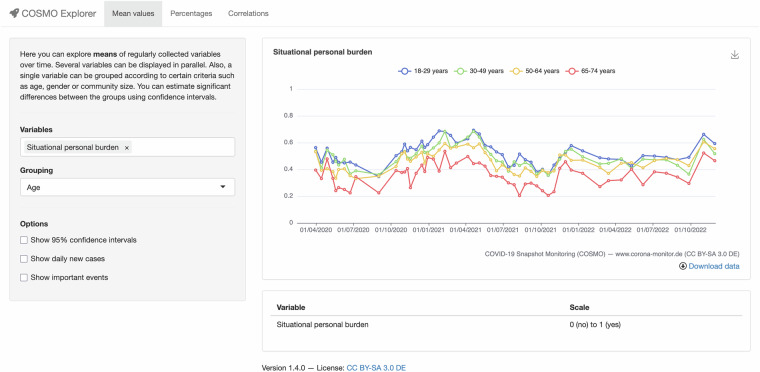
User interface for the COSMO Explorer. The interactive app allows users to investigate the development of variable means and distributions, as well as their correlations over time. *Major events: (1) first lockdown, 22.03.2020; (2) loosening of restrictions, 06.05.2020; (3) launch of Corona-Warning App, 16.06.2020; (4) first large protests in Berlin, 01.08.2020; (5) partial lockdown, 02.11.2020; (6) first vaccines undergoing approval, 15.11.2020; (7) lockdown, 16.12.2020; (8) first loosening of restrictions after lockdown, 01.03.2021; (9) 3 G regulations (access to public spaces when vaccinated, tested or recovered), 24.11.2021; (10) new government taking over, 08.12.2021; (11) expiration of 3 G regulations, 19.03.2021.

In order to explore the data in an interactive way, we also provide code for the COSMO Explorer (https://explore.healthpsychology.uni-bamberg.de/shiny/cosmo-explorer-en/), a web-based app that allows users to directly investigate variables (Fig. 1). The variables in the final version include risk perceptions, several protective behaviours, worries and fears, trust, attitudes towards several implemented and discussed measures and regulations, willingness to be vaccinated and several reasons for or against vaccination and belief in conspiracies. Details about the measurement of each variable (e.g., the measurement range) are directly described in a table below the visualisation. The recent version visualises:

1. The development of variable means over time (as shown in Fig. 1): The app allows users to examine how one or multiple variables unfold over time. Variables can be stratified according to various dimensions (as exemplified in Fig. 1), including demographics, such as age, gender, or education, as well as the acceptance of implemented response measures, allowing us to compare specific subgroups in the population. Confidence intervals allow users to estimate significant differences between timepoints and groups.
2. Development of variable distributions over time: The app visualizes the change of distributions for single variables. This information can help to better understand changes in mean values.
3. Correlations between variables: The app visualises relationships between variables by displaying the development of correlation coefficients over time (e.g., risk perception and acceptance of policies). While causation cannot be implied, information about the relationships between perceptions, attitudes and behaviours allows theory testing or development and can indicate important targets for future research questions, interventions and measures.

The development of variables and their correlations can be interpreted against the background of incidence rates and important pandemic events, which can also be displayed in the app (see Figure note; for more events consult respective repositories, e.g.^8^). The COSMO Explorer can be adapted to visualize COSMO data from other countries as well. The app is implemented in R using the Shiny framework. Researchers from other countries are invited to use the code and add other variables included in the dataset to the explorer.

### Limitations

The urgency of the situation incurs some limitations to the study, including limited opportunities for scientific review and validation, as described above. This was also pointed out in the WHO standard protocol that was developed based on this work^11^.

## Data Availability

The code used to produce the shiny app (COSMO Explorer) is openly available on GitLab (https://gitlab.com/pidilab/cosmo-explorer) and can be used under a CC-BY-NC license.
